# 云南省肿瘤医院2013-2022年15,967例肺癌手术患者临床流行病学特征分析

**DOI:** 10.3779/j.issn.1009-3419.2024.101.33

**Published:** 2024-12-20

**Authors:** Ruke TANG, Yujie LEI, Lianhua YE, Guangqiang ZHAO, Xudong XIANG, Gaofeng LI, Guangjian LI, Xi WANG, Ying CHEN, Kaiyun YANG, Xiaobo CHEN, Jiapeng YANG, Min ZHAO, Bingquan XIANG, Qiubo HUANG, Guangcan LUO, Hongwei ZHANG, Yunchao HUANG

**Affiliations:** ^1^650118 昆明，云南省肿瘤医院胸外一科; ^1^Department of Thoracic Surgery I, Yunnan Cancer Hospital, Kunming 650118, China; ^2^650118 昆明，云南省胸外二科; ^2^Department of Thoracic Surgery II, Yunnan Cancer Hospital, Kunming 650118, China; ^3^650118 昆明，云南省医务部; ^3^Department of Medical Administration, Yunnan Cancer Hospital, Kunming 650118, China; ^4^650118 昆明，云南省重症医学科; ^4^Department of Intensive Care Unit, Yunnan Cancer Hospital, Kunming 650118, China

**Keywords:** 肺肿瘤, 手术, 流行病学, 病理类型, 云南, Lung neoplasms, Surgery, Epidemiology, Pathological type, Yunnan

## Abstract

**背景与目的:**

云南省肺癌呈现区域性高发，目前鲜有云南省肺癌临床流行病学相关的大样本研究。本研究探究近10年于云南省肿瘤医院行肺癌手术治疗患者的流行病学特点，旨在为肺癌的防治工作提供理论参考。

**方法:**

收集2013-2022年在云南省肿瘤医院行手术治疗的15,967例肺癌患者的临床资料，对患者的一般资料、手术信息、肺癌病理分型等临床流行病学特点进行统计分析。

**结果:**

15,967例肺癌病例中，男性占46.3%，女性占53.7%，2013-2022年男女性别比例在0.68-1.61:1之间波动。年龄的中位数56（49, 63）岁，50-59岁年龄段患者占37.0%，2017年以来，60岁以下患者占比逐年增长。吸烟患者28.1%，未吸烟患者71.9%。曲靖市（41.4%）和昆明市（23.2%）是云南省病例数分布前两位的市，29.6%的云南省患者来自曲靖市的宣威、富源地区。右肺上叶占28.2%、右肺中叶占6.3%、右肺下叶占20.1%、左肺上叶占22.7%、左肺下叶占16.4%。胸腔镜手术占比从30.8%增加至96.3%，单孔胸腔镜手术占61.3%。肺叶切除术占64.2%，肺楔形切除术占17.2%，肺段切除术占12.2%，肺叶切除术从83. 1%下降至46.1%。0-I期从43.5%上升至82.8%，II-IV期从56.5%下降至17.2%。腺癌从75.6%增加至88.3%，鳞癌从21.5%下降至8.6%；腺癌中女性占60.9%，鳞癌中男性占90.6%；腺癌就诊年龄高峰为50-59岁，鳞癌就诊年龄高峰为60-69岁；鳞癌患者吸烟率为65.9%，腺癌患者吸烟率为22.3%；腺癌患者0-I期占76.3%，鳞癌患者II-III期占64.1%。

**结论:**

女性腺癌患者比例增加；发病年龄呈现年轻化；非吸烟肺癌患者比例增加； 0-I期肺癌比例增加等可能是近10年云南及周边地区肺癌手术患者流行病学特征的变化趋势。

## Abstract (en)

**Background and objective:**

Lung cancer is a disease with a high incidence rate in Yunnan province, yet there is a paucity of large-scale studies on its clinical epidemiology. This research aims to investigate the epidemiological characteristics of patients who underwent lung cancer surgery at Yunnan Cancer Hospital over the past decade, thereby providing a theoretical basis for the prevention and treatment of lung cancer.

**Methods:**

Clinical data were collected from 15,967 patients who underwent lung cancer surgery at Yunnan Cancer Hospital between 2013 and 2022. A statistical analysis was conducted on the patients' general data, surgical information, pathological types of lung cancer, and other clinical epidemiological characteristics.

**Results:**

Among the 15,967 cases of lung cancer, 46.3% were male and 53.7% were female, with the male-to-female ratio ranging from 0.68 to 1.61:1. The median age was 56 years (interquartile range: 49-63), and 37.0% of the patients were in the age group of 50-59 years. Since 2017, there has been an annual increase in the proportion of patients under the age of 60 years. The smoking status of the patients showed that 28.1% were smokers and 71.9% were non-smokers. Qujing city accounted for 41.4% and Kunming city for 23.2% of the cases in Yunnan province, with 29.6% of patients originating from Xuanwei and Fuyuan areas of Qujing city. The distribution of affected lung lobes was as follows: right upper lobe 28.2%, right middle lobe 6.3%, right lower lobe 20.1%, left upper lobe 22.7%, and left lower lobe 16.4%. The use of thoracoscopic surgery increased from 30.8% to 96.3%, with single-port thoracoscopic surgery comprising 61.3%. Lobectomy was performed in 64.2% of cases, wedge resection in 17.2%, and segmentectomy in 12.2%. The proportion of lobectomy decreased from 83.1% to 46.1%. The proportion of patients in stages 0-I increased from 43.5% to 82.8%, while stages II-IV decreased from 56.5% to 17.2%. Adenocarcinoma increased from 75.6% to 88.3%, and squamous cell carcinoma decreased from 21.5% to 8.6%. Among adenocarcinoma patients, 60.9% were female. Among sguamous cell carcinoma patients, 90.6% were male. The peak age for adenocarcinoma was 50-59 years, and for squamous cell carcinoma, it was 60-69 years. The smoking rate was higher among squamous cell carcinoma patients (65.9%) compared to adenocarcinoma patients (22.3%). Adenocarcinoma patients had a higher proportion in stages 0-I (76.3%), while squamous cell carcinoma patients were more prevalent in stages II-III (64.1%).

**Conclusion:**

The findings indicate an increasing proportion of female patients with adenocarcinoma, a younger age of onset, a higher proportion of non-smoking lung cancer patients, and an increased proportion of stages 0-I lung cancer. These trends may reflect the epidemiological characteristics of patients undergoing lung cancer surgery in Yunnan and surrounding areas over the past decade.

肺癌是一种多步骤、多因素造成的病因不明疾病，吸烟、空气污染、职业暴露、饮食习惯、家族遗传和肺部基础疾病等都在肺癌的发展中发挥重要作用^[1]^。肺癌是当今全球死亡率最高的恶性肿瘤^[2]^。随着人口老龄化，中国的癌症负担不断加重。肺癌的发病率和死亡率在中国的恶性肿瘤中均占到第一^[3]^，在过去10年呈上升趋势^[4]^。

近年来，肺癌在基础研究、预防、诊断、手术和多学科治疗等领域都取得了重要进展。肺癌的流行病学和病理学特征也发生了变化。据报道^[5⇓-7]^，女性和从不吸烟者的肺癌发病率上升。肺癌的患病年龄也在发生变化。根据国家癌症中心（National Cancer Center, NCC）统计的肺癌患者10年的数据，年龄≥60岁的患者比例由2005年的41.2%上升至2014年的56.2%，年龄<60岁的患者比例呈下降趋势^[8]^。研究^[5,9]^表明腺癌的发病率在上升，鳞癌的发病率在下降，腺癌目前已成为肺癌中发病率第一的病理类型。

云南省位于我国的西南部，肺癌发病呈现区域性高发，尤其是在曲靖市的宣威、富源地区^[10]^。目前鲜有云南省肺癌临床流行病学相关的大样本研究，对于社会人口学、手术和病理特征的变化情况未知。本研究旨在对云南省肿瘤医院行手术治疗的肺癌患者的临床流行病学特征进行描述性分析，观察近10年人群、手术和病理类型特征的变化情况。

## 1 资料与方法

### 1.1 研究对象

纳入2013至2022年云南省肿瘤医院15,967例肺癌手术患者。（1）纳入标准：2013年1月1日至2022年12月31日在云南省肿瘤医院行手术治疗；经手术或穿刺、纤维支气管镜活检等检查取得明确的病理诊断；病理诊断明确为原发性肺癌。（2）排除标准：临床或病理资料不完整；多次接受手术的同一患者，仅纳入首次接受手术治疗的临床资料。本研究已获云南省肿瘤医院伦理委员会批准，本研究中的所有程序均遵循2013年修订的《赫尔辛基宣言》的原则，纳入患者资料见图1。

**图 1:**
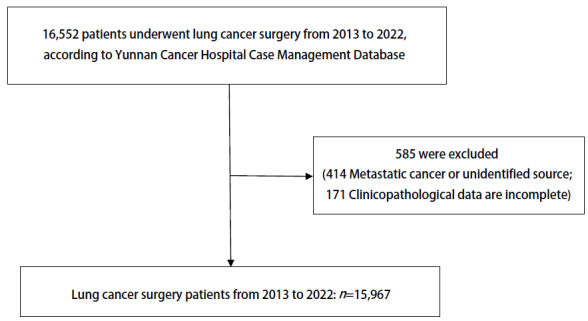
研究对象的入选流程图

### 1.2 临床资料

来源于云南省肿瘤医院病案管理数据库。包括性别、年龄、出生地、自我报告吸烟史、发病部位、手术情况、肿瘤原发灶-淋巴结-转移（tumor-node-metastasis, TNM）分期及病理类型等。吸烟定义为每天至少吸1支烟、至少连续6个月^[11]^。

### 1.3 TNM分期及病理分型

TNM分期标准：采用美国癌症联合委员会的第七版和第八版TNM分期标准，分为0（原位癌TisN0M0）、I、II、III、IV期。诊断中未分期的病例，由研究者根据病理报告自行分期。肺癌病理分型标准：依据第五版《世界卫生组织胸部肿瘤分类》（2021版分类）^[12]^规定，通过国际疾病分类（International Classification of Diseases, ICD）-O编码中标记为“2、3”认为是原发性恶性肿瘤。具体分为鳞癌、腺癌、小细胞癌、大细胞癌和其他类型5组，其他类型包括腺鳞癌、肉瘤样癌、类癌等。原位腺癌（adenocarcinoma in situ, AIS）归为腺癌，原位鳞癌归为鳞癌。

### 1.4 统计学方法

应用SPSS 26.0进行统计描述和统计学分析，计量资料以中位数和四分位数间距表示。计数资料用频数和构成比表示，采用卡方检验或Fisher确切概率法比较组间差异。*P*<0.05为差异有统计学意义。

## 2 结果

2013-2022年就诊于云南省肿瘤医院，病理确诊为原发性肺癌的手术患者共15,967例。其中2013年591例，2014年687例，2015年731例，2016年839例，2017年914例，2018年1366例，2019年1894例，2020年2313例，2021年3192例，2022年3440例。10年来肺癌手术病例数逐年上升，2013-2017年增长相对缓慢，2018-2022年增长飞快。

### 2.1 社会人口学特征

15,967例肺癌手术患者中，男性7389例（46.3%），女性8578例（53.7%）。2013-2022年原发性肺癌手术患者的男女性别比例在0.68-1.61:1之间波动，2017年及以前男性患者数量高于女性，之后女性数量都高于男性（图2A）。患者最大年龄86岁，最小年龄7岁，年龄中位数56（49, 63）岁。50-59岁年龄段占比最高（37.0%），<30岁和≥80岁占比较低（共占1.3%）。2017年以来，<60岁的患者占比在逐年增长，≥60岁患者占比逐年下降（图2B）。15,939例患者能获得吸烟史，吸烟病例共4483例（28.1%），其中男性吸烟患者4367例（59.2%），女性吸烟患者116例（1.4%）。吸烟患者占比从2013年的33.5%下降至2022年的21.2%（图2C）。15,967例中，云南省14,842例，贵州省795例，其他省份330例。14,842例云南肺癌患者中，曲靖市6145例（41.4%）和昆明市3448例（23.2%）相对较多。曲靖市宣威、富源地区共4391例，占29.6%。

**图 2:**
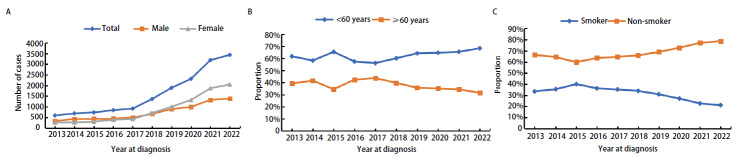
社会人口学特征。A：不同年份的病例数变化；B：不同年份的年龄变化；C：不同年份的吸烟史变化。

### 2.2 临床手术特征

右肺肺癌共9354例（58.6%），左肺肺癌共6480例（40.6%），双肺肺癌共133例（0.8%）。单一肺叶病变分布分别为：右肺上叶（28.2%）、右肺中叶（6.3%）、右肺下叶（20.1%）、左肺上叶（22.7%）、左肺下叶（16.4%）。

开胸手术的数量占比从2013年的69.2%减少到2022年的3.7%，胸腔镜手术数量占比则是从2013年的30.8%增加到2022年的96.3%。单孔胸腔镜手术9793例（61.3%），三孔胸腔镜手术2330例（14.6%）。2013-2016年以三孔胸腔镜为主，2017年单孔胸腔镜和单操作孔胸腔镜增长率较高，2017年及以后单孔胸腔镜成为最常用的手术技术（图3A）。10,245例（64.2%）患者进行了肺叶切除术，2745例（17.2%）患者进行了肺楔形切除术，1944例（12.2%）患者进行了肺段切除术，1033例（6.4%）患者接受了涉及多个肺叶的手术（袖式、全肺、楔形/肺段/肺叶中的至少两种）。10年来，肺叶切除术的比例在逐渐降低，亚肺叶切除术（楔形+肺段）的比例在逐渐增加（图3B）。15,961例患者能获得具体的TNM分期，I期肺癌患者共10,191例（63.8%），III期肺癌患者共2240例（14.0%），II期肺癌患者共1832例（11.5%），0期肺癌患者共1004例（6.4%），IV期肺癌患者共694例（4.3%）。10年间，0-I期患者的比例逐渐上升，II-IV期患者的比例逐渐下降（图3C）。

**图 3:**
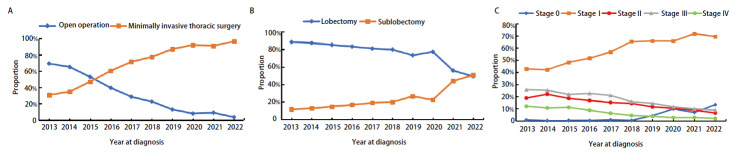
临床手术特征。A：不同年份的手术技术变化；B：不同年份的手术方式变化；C：不同年份的TNM分期变化。

### 2.3 病理类型特征

病理类型比例从高到低分别为腺癌（84.3%）、鳞癌（11.9%）、小细胞癌（0.7%）和大细胞癌（0.3%）。其他病理类型包括肉瘤样癌、腺鳞癌、类癌、腺样囊性癌等，共占2.8%。10年间腺癌比例从2013年的75.6%增长到2022年的88.3%，鳞状细胞癌比例从2013年的21.5%下降到2022年的8.6%（图4A）。腺癌中，男性占39.1%，女性占60.9%。鳞癌中，男性占90.6%，女性占9.4%。男女性中的腺癌占比都超过总体的一半，腺癌中女性比例比男性高，鳞癌中男性比例高于女性（9.64:1）（图4B）。腺癌在30-39岁之间的占比最高，就诊年龄高峰在50-59岁，随着年龄的增长腺癌的占比在下降。鳞癌在70-79岁之间的占比最高，就诊年龄高峰在60-69岁。无论任何年龄段，腺癌的病例数始终高于其他任何病理类型（图4C）。吸烟患者中鳞癌比例（27.5%）高于不吸烟患者中的鳞癌比例（5.8%），腺癌比例（66.8%）低于不吸烟患者中的腺癌比例（91.2%）（图4D）。相较其他分期中的患者，腺癌在0期和I期中占比（76.3%）相对更高，鳞癌在II期和III期中占比（64.1%）相对更高（图4E）。

**图 4:**
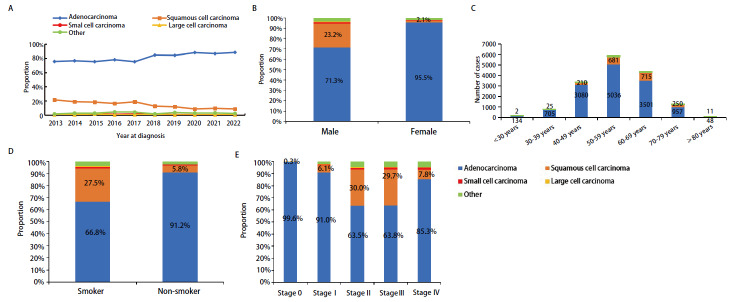
病理类型特征。A：不同年份的病理类型变化；B：男女病理类型构成分布图；C：不同年龄病理类型构成分布图；D：吸烟者和非吸烟者病理类型构成分布图；E：不同TNM分期病理类型构成分布图。

## 3 讨论

本研究对云南省肿瘤医院2013-2022年肺癌手术患者的临床流行病学特征进行了分析。结果显示，2013-2022年在云南省肿瘤医院接受手术治疗的肺癌患者人数逐年持续增加，2022年接受手术治疗的肺癌新发病例数量为2013年的5.82倍。提示与肺癌的筛查和早诊早治开展以及我国肺癌发病率的逐年升高息息相关。

本研究结果显示，腺癌、鳞癌、小细胞癌和大细胞癌所占比例分别为84.3%、11.9%、0.7%和0.3%，与北京^[13]^、辽宁^[14]^、四川^[15]^等地区相关文献报道相似。腺癌比例增长，鳞癌比例下降，与国内6个省份16家医院开展的一个多中心研究^[16]^的结论一致。腺癌中女性比例比男性高，鳞癌中男性比例远高于女性，研究结果与北京、辽宁地区的相关研究结果^[13,14]^一致。女性肺腺癌的大量增加可能有以下原因：（1）云南省吸烟人群基数大，公共场所及家庭中的二手烟暴露常常导致女性被动吸烟；（2）云南省部分农村地区妇女用当地烟煤做饭和取暖，烟煤燃烧引起的空气污染（包括多环芳烃、细颗粒物、纳米级二氧化硅等）可能导致肺癌的发生^[17,18]^。

年龄是肺癌的主要发病因素之一^[19]^。2017年以来60岁以下的患者数量占比在逐年增长，中青年肺癌患者占比增加，提示肺癌患者逐渐趋向年轻化，与国内部分研究报道^[14,16]^相符合，与NCC统计2005-2014年的结论^[8]^相反。可能与越来越多的早期肺癌患者得到了手术治疗，人群的健康体检意识在不断提高有关。云南已于2002年进入人口老龄化社会，且人口老龄化程度在不断加深，部分老年人因健康意识缺乏，在肺癌发病高峰年龄未及时确诊，直到肺癌进展出现症状再接受治疗，这类患者的治疗过程往往会消耗更多医疗资源，这将会进一步加重我国的肺癌负担，所以中老年人应该受到更多的关注，尽早开始肺癌筛查以及定期随访。腺癌就诊年龄高峰在50-59岁，鳞癌高峰在60-69岁，与既往相关研究结论^[16]^一致。有研究^[20,21]^显示，宣威地区的家族遗传和遗传易感可能会导致腺癌的发病更加年轻。本研究中65.9%鳞癌患者吸烟，香烟烟雾的长期刺激可能导致左右主支气管及叶支气管鳞状上皮增生，进而导致鳞癌，且肺部基础疾病及免疫力低下都可能导致鳞癌的发生^[22]^，因此鳞癌的高峰略晚于腺癌。

吸烟是导致肺癌的主要原因。本研究中，约65.9%鳞癌患者吸烟，腺癌则更常见于不吸烟者，约77.7%腺癌患者不吸烟，本研究结果与云南^[23]^、四川^[24]^、河南^[25]^、辽宁^[14]^等地区研究结果相一致。云南省作为中国烟草的主要种植基地，人群总体吸烟率高于全球和中国的成人吸烟率^[26]^。近年来，在国家大力开展戒烟教育和控烟活动的作用下，人群总体吸烟率在下降^[27]^。本研究中的肺癌患者吸烟比例逐年降低，提示云南省肺癌人群吸烟患者越来越少，教育作用取得了初步成效，但烟草控制仍是中国肺癌预防和控制的最重要问题之一。

2020年全国第七次人口普查数据^[28]^显示，云南省总人口约为4720万，人口居前两位的是昆明市846万（17.9%）和曲靖市576万（12.2%）。在本研究中，曲靖市病例数最多（41.4%），其次是昆明市（23.2%）。其他地州市病例数和人口数比例几乎一致。对于昆明市病例分布的差异，原因可能如下：云南省肿瘤医院位于昆明市，医疗卫生资源丰厚，昆明周边地区患者就诊方便，同时城市癌症早诊早治项目在昆明长期开展^[29]^，昆明市常住人口健康意识强。曲靖市的宣威、富源地区肺癌发病率、死亡率在全世界较高^[30]^。宣威、富源地区人口占到云南省的4.0%^[28]^，本研究中病例数占云南省29.6%。宣威、富源地区是农村高发肺癌早诊早治的工作试点，随着各级政府在宣威地区开展肺癌筛查和早诊早治，当地群众对肺癌重视并积极进行健康体检，大量的年轻肺癌患者体检和筛查出高危肺结节后尽早进行手术治疗。云南省肿瘤医院5.0%的肺癌手术病例来自于贵州，贵州省六盘水市紧邻云南省宣威、富源地区，肺癌发病情况有很多的相似之处，加之云南省在对宣威肺癌综合防治方面具有先进性，因此该地区患者更倾向于至云南省就诊。

在本研究中，10年间电视辅助胸腔镜手术（video-assisted thoracic surgery, VATS）数量占比则是从30.8%增加至96.3%，这种手术趋势的变化与中国医科大学第一附属医院的报道相符^[14]^。既往臧若川等^[31]^开展的多中心大样本研究发现，VATS的比例在10年间显著增加（从2005年的0.6%增加到2014年的34.4%）。20年来，VATS得到了快速发展。本研究中，10年间单孔胸腔镜手术从0例（0.0%）增加至3214例（93.5%），目前已成为各类型肺癌根治术中最常用的技术，这种手术的趋于成熟让更多的患者成功地得到了微创治疗，最大限度地减少了手术对患者身体和心理的伤害。

本研究中，肺叶切除术的比例在逐年降低，亚肺叶切除术的比例在逐年增加，研究结果与国内的多中心研究结果^[32]^基本符合，肺叶切除术在临床中更常使用，但随着0、I期病例的增多，综合多方面因素全面评估，逐年增多的亚肺叶切除术成功开展让患者的创伤减小和多原发肺癌二次手术打下基础，真正意义上实现了早诊早治。

NCC发布过肺癌患者2005-2014年的数据^[8]^，I期肺癌比例从17.2%增长到21.1%，而II-IIIA期肺癌的比例从 41.9%降低到31.5%。本研究显示，手术肺癌患者中，0-I期肺癌比例从43.5%增长至82.8%，II-III期肺癌的比例从44.5%降低到15.3%。可以看出0、I期肺癌比例快速上升，同时II、III期肺癌比例快速下降，与NCC公布的数据呈现出一致性的变化趋势，但是本研究的研究对象是手术患者，因此两者在比例上会有所差异。日本肺癌登记数据库公布的手术肺癌分期数据^[33]^也与本研究的结果相符。本研究还发现，腺癌患者在0期（99.6%）和I期（91.0%）中占比相对更高，鳞癌患者在II期（30.0%）和III期（29.7%）中占比相对更高，本研究结果与四川大学华西医院的研究结果相似^[34]^。随着低剂量螺旋计算机断层扫描的普及^[35]^，AIS、微浸润腺癌的发现率逐渐上升，这些患者尽早得到了手术治疗，0、I期腺癌的比例因此不断上升。鳞癌相对其他病理类型中央型较多，肿物较大，常生长于支气管中，更容易发生淋巴结转移，II、III期鳞癌的比例因此相对更高。

本研究首次报告了2013-2022年云南省肿瘤医院15,967例肺癌手术患者的临床流行病学特征及变化趋势。研究发现近年来女性腺癌患者比例增加、发病年龄呈现年轻化、非吸烟肺癌患者比例增加、0-I期肺癌比例增加等可能是近10年云南及周边地区肺癌手术患者流行病学特征的变化趋势。但是由于仅纳入单中心数据，加之患者的就诊行为受地域影响较大，因此本研究仅能代表云南省的部分病例情况。除上述外，不可排除经济文化水平、环境污染、生活习惯等相关因素对研究结果产生的偏倚。未来多中心、大样本研究提供更全面的云南省肺癌流行病学资料，才能科学地制定云南省肺癌防治政策。同时，对于肺癌手术患者的社会人口学、手术和病理特征等因素变化的生存预后分析值得下一步研究。
